# Identification of six *Cytospora* species on Chinese chestnut in China

**DOI:** 10.3897/mycokeys.62.47425

**Published:** 2020-01-13

**Authors:** Ning Jiang, Qin Yang, Xin-Lei Fan, Cheng-Ming Tian

**Affiliations:** 1 The Key Laboratory for Silviculture and Conservation of the Ministry of Education, Beijing Forestry University Beijing China; 2 Beijing Forestry University, Beijing 100083, China Central South University of Forestry and Technology Changsha China; 3 Forestry Biotechnology Hunan Key Laboratories, Central South University of Forestry and Technology, Changsha 410004, China Beijing Forestry University Beijing China; 4 Hunan Key Laboratory of Forest Protection, Central South University of Forestry and Technology, Changsha 410004, China Central South University of Forestry and Technology Changsha China

**Keywords:** *Castanea mollissima*, Cytosporaceae, Diaporthales, systematics, taxonomy

## Abstract

Chinese chestnut (*Castanea mollissima*) is an important crop tree species in China. In the present study, *Cytospora* specimens were collected from Chinese chestnut trees and identified using molecular data of combined ITS, LSU, ACT and RPB2 loci, as well as morphological features. As a result, two new *Cytospora* species and four new host records were confirmed, viz. *C. kuanchengensis***sp. nov.**, *C. xinglongensis***sp. nov.**, *C. ceratospermopsis*, *C. leucostoma*, *C. myrtagena* and *C. schulzeri*.

## Introduction

Chinese chestnut (*Castanea mollissima*) is a widely cultivated crop tree species in China, producing nutritious and delicious nuts for humans (Lu and Guo 2016). However, *Cryphonectria parasitica* and several fungi are causing severe chestnut diseases worldwide, which reduce the nut production, even killing the hosts. (Aghayeva et al. 2017, Shuttleworth and Guest 2017, Jiang et al. 2018a, Rigling and Prospero 2018). Recently, several diaporthalean species were described from Chinese chestnut trees for the clear taxonomic concepts of families, genera and species in Diaporthales (Rossman et al. 2007, Senanayake et al. 2017, 2018), including species of *Aurantiosacculus*, *Coryneum*, *Cryphonectria*, *Dendrostoma*, *Endothia*, *Gnomoniopsis*, *Neopseudomelanconis* and *Ophiognomonia* (Gong et al. 2017, Jiang et al. 2018b, 2018c, 2019a, 2019b, Jiang and Tian 2019).

*Cytospora* (Cytosporaceae, Diaporthales) is a widely distributed genus worldwide, occurring on a broad range of hosts (Sarma and Hyde 2001, Yang et al. 2015, Lawrence et al. 2017, Norphanphoun et al. 2017, 2018, Wijayawardene et al. 2018, Jayawardena et al. 2019, Phookamsak et al. 2019, Fan et al. 2020). Some species can cause severe canker diseases on woody trees, such as *Cytospora chrysosperma*, which is a commom pathogen on the commercial tree genera, *Populus* and *Salix* (Fan et al. 2014b, Zhang et al. 2014, Kepley et al. 2015, Wang et al. 2015). Host affiliation was considerd as the main evidence for separating species in *Cytospora* before DNA sequences were used; however, morphology combined with phylogeny has revealed many cryptic species. For example, 28 *Cytospora* species were discovered from *Eucalyptus* from South Africa (Adams et al. 2005) and six from apple trees in Iran (Mehrabi et al. 2011), three from Chinese scholar tree (Fan et al. 2014a), four from walnut tree (Fan et al. 2015a), six from anti-desertification plants in China (Fan et al. 2015b) and two from grapevine in North America (Lawrence et al. 2017). Several recent studies discovered new species of *Cytospora* using multiphasic analyses (Lawrence et al. 2018, Norphanphoun et al. 2017, 2018, Senanayake et al. 2017, 2018, Pan et al. 2018, Zhu et al. 2018, Zhang et al. 2019).

**Figure 1.**
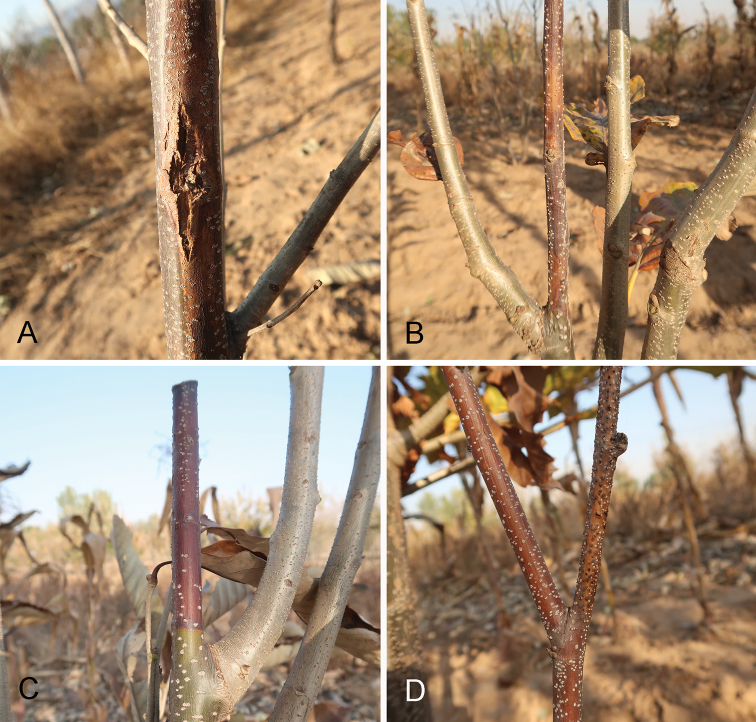
Canker symptoms on *Castanea mollissima* caused by *Cytospora* spp.

During our investigations of chestnut disease in China from 2016 to 2019, diseased branches with typical *Cytospora* fruiting bodies were discovered and collected (Fig. 1). In the present study, *Cytospora* species from *Castanea mollissima* were identified using a combined method of morphology and phylogeny.

## Materials and methods

### Sample collections and isolations

Chinese chestnut has a wide distribution in China. In the present study, we surveyed Hebei, Shaanxi and Shandong Provinces from 2016 to 2019. Dead and dying branches with typical *Cytospora* fruiting bodies were collected and packed in paper bags. Isolates were obtained by removing the ascospores or conidial masses from the fruiting bodies on to clean PDA plates and incubating at 25 °C until spores germinated. Single germinated spores were transferred on to the new PDA plates and incubated at 25 °C in the dark. Specimens were deposited in the Museum of the Beijing Forestry University (BJFC) and axenic cultures are maintained in the China Forestry Culture Collection Centre (CFCC).

### Morphological analysis

Observation and description of *Cytospora* species from *Castanea mollissima* was based on fruiting bodies formed on tree barks. Ascomata and conidiomata from tree barks were sectioned by hand using a double-edged blade and strctures were observed under a dissecting microscope. At least 10 conidiostromata/ascostromata, 10 asci and 50 conidia/ascospores were measured to calculate the mean size and standard deviation. Measurements are reported as maximum and minimum in parentheses and the range representing the mean plus and minus the standard deviation of the number of measurements is given in parentheses (Voglmayr et al. 2017). Microscopy photographs were captured with a Nikon Eclipse 80i compound microscope equipped with a Nikon digital sight DS-Ri2 high definition colour camera, using differential interference contrast illumination. Introduction of the new species, based on molecular data, follow the recommendations of Jeewon and Hyde (2016).

### DNA extraction, PCR amplification and sequencing

Genomic DNA was extracted from young mycelium growing on PDA plates following Doyle and Doyle (1990). PCR amplifications were performed in a DNA Engine Peltier Thermal Cycler (PTC-200; Bio-Rad Laboratories, Hercules, CA, USA). The primer pair ITS1/ITS4 (White et al. 1990) was used to amplify the ITS region. The primer pair LR0R/LR5 (Vilgalys and Hester 1990) was used to amplify the LSU region. The primer pair ACT512F/ACT783R (Carbone and Kohn 1999) was used to amplify ACT gene. The primer pair dRPB2-5f/dRPB2-7r (Voglmayr et al. 2016) was used to amplify the RPB2 gene. The polymerase chain reaction (PCR) assay was conducted as described in Fan et al. (2020). PCR amplification products were assayed via electrophoresis in 2% agarose gels. DNA sequencing was performed using an ABI PRISM 3730XL DNA Analyzer with a BigDye Terminater Kit v.3.1 (Invitrogen, USA) at the Shanghai Invitrogen Biological Technology Company Limited (Beijing, China).

### Phylogenetic analyses

The preliminary identities of the isolates sequenced were obtained by conducting a standard nucleotide BLAST search using ITS, LSU, ACT and RPB2. Then all *Cytospora* isolates were selected to conduct phylogenetic analyses, based on sequence datasets from Fan et al. (2020). *Diaporthe vaccinia* (CBS 160.32) in Diaporthaceae was selected as the outgroup taxon. All sequences were aligned using MAFFT v. 6 (Katoh and Toh 2010) and edited manually using MEGA v. 6 (Tamura et al. 2013). Phylogenetic analyses were performed using PAUP v. 4.0b10 for Maximum Parsimony (MP) analysis (Swofford 2003) and PhyML v. 3.0 for Maximum Likelihood (ML) analysis (Guindon et al. 2010).

MP analysis was run using a heuristic search option of 1000 search replicates with random-additions of sequences with a tree bisection and reconnection algorithm. Maxtrees were set to 5000, branches of zero length were collapsed and all equally parsimonious trees were saved. Other calculated parsimony scores were tree length (TL), consistency index (CI), retention index (RI) and rescaled consistency (RC). ML analysis was performed using a GTR site substitution model including a gamma-distributed rate heterogeneity and a proportion of invariant sites (Guindon et al. 2010). The branch support was evaluated using a bootstrapping method of 1000 replicates (Hillis and Bull 1993). Phylograms were shown using FigTree v. 1.4.3 (Rambaut 2016). Novel sequences, generated in the current study, were deposited in GenBank (Table 1) and the aligned matrices used for phylogenetic analyses in TreeBASE (accession number: S25160).

**Table 1.** Strains used in the phylogenetic tree and their culture accession and GenBank numbers. Strains from this study are in bold and ex-strains are marked with *.

Species	Strain	Host	Origin	GenBank accession numbers			
				ITS	LSU	ACT	RPB2
*Cytospora ailanthicola*	CFCC 89970*	*Ailanthus altissima*	China	MH933618	MH933653	MH933526	MH933592
*Cytospora abyssinica*	CMW 10181*	*Eucalyptus globulus*	Ethiopia	AY347353	NA	NA	NA
*Cytospora acaciae*	CBS 468.69	*Ceratonia siliqua*	Spain	DQ243804	NA	NA	NA
*Cytospora ampulliformis*	MFLUCC 16-0583*	*Sorbus intermedia*	Russia	KY417726	KY417760	KY417692	KY417794
	MFLUCC 16-0629	*Acer platanoides*	Russia	KY417727	KY417761	KY417693	KY417795
*Cytospora amygdali*	CBS 144233*	*Prunus dulcis*	USA	MG971853	NA	MG972002	NA
	CFCC 89615	*Juglans regia*	China	KR045618	KR045700	KF498673	KU710946
	CFCC 89616	*Juglans regia*	China	KR045619	KR045701	KF498674	KU710947
*Cytospora atrocirrhata*	CFCC 89615	*Juglans regia*	China	KR045618	KR045700	KF498673	KU710946
*Cytospora austromontana*	CMW 6735*	*Eucalyptus pauciflora*	Australia	AY347361	NA	NA	NA
*Cytospora beilinensis*	CFCC 50493*	*Pinus armandii*	China	MH933619	MH933654	MH933527	NA
	CFCC 50494	*Pinus armandii*	China	MH933620	MH933655	MH933528	NA
*Cytospora berberidis*	CFCC 89927*	*Berberis dasystachya*	China	KR045620	KR045702	KU710990	KU710948
	CFCC 89933	*Berberis dasystachya*	China	KR045621	KR045703	KU710991	KU710949
*Cytospora berkeleyi*	StanfordT3*	*Eucalyptus globulus*	USA	AY347350	NA	NA	NA
	UCBTwig3	*Eucalyptus globulus*	USA	AY347349	NA	NA	NA
*Cytospora brevispora*	CBS 116811*	*Eucalyptus grandis × tereticornis*	Congo	AF192315	NA	NA	NA
	CBS 116829	*Eucalyptus grandis*	Venezuela	AF192321	NA	NA	NA
*Cytospora bungeanae*	CFCC 50495*	*Pinus bungeana*	China	MH933621	MH933656	MH933529	MH933593
	CFCC 50496	*Pinus bungeana*	China	MH933622	MH933657	MH933530	MH933594
*Cytospora californica*	CBS 144234*	*Juglans regia*	USA	MG971935	NA	MG972083	NA
*Cytospora carbonacea*	CFCC 89947	*Ulmus pumila*	China	KR045622	KP310812	KP310842	KU710950
*Cytospora carpobroti*	CMW 48981*	*Carpobrotus edulis*	South Africa	MH382812	MH411216	NA	NA
*Cytospora castaneae*	AUCCT/DBT 183*	*Castanea sativa*	India	KC963921	NA	NA	NA
*Cytospora cedri*	CBS 196.50	NA	Italy	AF192311	NA	NA	NA
*Cytospora celtidicola*	CFCC 50497*	*Celtis sinensis*	China	MH933623	MH933658	MH933531	MH933595
	CFCC 50498	*Celtis sinensis*	China	MH933624	MH933659	MH933532	MH933596
*Cytospora centrivillosa*	MFLUCC 16-1206*	*Sorbus domestica*	Italy	MF190122	MF190068	NA	MF377601
	MFLU 17-0887	*Sorbus domestica*	Italy	MF190123	MF190069	NA	NA
	MFLUCC 17-1660	*Sorbus domestica*	Italy	MF190124	MF190070	NA	MF377600
*Cytospora ceratosperma*	CFCC 89624	*Juglans regia*	China	KR045645	KR045724	NA	KU710976
	CFCC 89625	*Juglans regia*	China	KR045646	KR045725	NA	KU710977
***Cytospora ceratospermopsis***	CFCC 89626*	*Juglans regia*	China	KR045647	KR045726	KU711011	KU710978
	CFCC 89627	*Juglans regia*	China	KR045648	KR045727	KU711012	KU710979
	**CFCC 52471**	***Castanea mollissima***	**China**	**MK432629**	**MK429899**	**MK442953**	**MK578087**
	**CFCC 52472**	***Castanea mollissima***	**China**	**MK432630**	**MK429900**	**MK442954**	**MK578088**
*Cytospora chrysosperma*	CFCC 89629	*Salix psammophila*	China	KF765673	KF765689	NA	KF765705
	CFCC 89981	Populus alba subsp. pyramidalis	China	MH933625	MH933660	MH933533	MH933597
	CFCC 89982	*Ulmus pumila*	China	KP281261	KP310805	KP310835	NA
*Cytospora cinerostroma*	CMW 5700*	*Eucalyptus globulus*	Chile	AY347377	NA	NA	NA
*Cytospora cotini*	MFLUCC 14-1050*	*Cotinus coggygria*	Russia	KX430142	KX430143	NA	KX430144
*Cytospora curvata*	MFLUCC 15-0865*	*Salix alba*	Russia	KY417728	KY417762	KY417694	KY417796
*Cytospora davidiana*	CXY 1350*	*Populus davidiana*	China	KM034870	NA	NA	NA
	CXY 1374	*Populus davidiana*	China	KM034869	NA	NA	NA
*Cytospora diatrypelloidea*	CMW 8549*	*Eucalyptus globulus*	Australia	AY347368	NA	NA	NA
*Cytospora disciformis*	CMW 6509*	*Eucalyptus grandis*	Uruguay	AY347374	NA	NA	NA
	CMW 6750	*Eucalyptus globulus*	Australia	AY347359	NA	NA	NA
*Cytospora donetzica*	MFLUCC 15-0864	NA	NA	KY417729	KY417763	KY417695	KY417797
	MFLUCC 16-0574*	*Rosa* sp.	Russia	KY417731	KY417764	KY417696	KY417798
*Cytospora elaeagni*	CFCC 89632	*Elaeagnus angustifolia*	China	KR045626	KR045706	KU710995	KU710955
	CFCC 89633	*Elaeagnus angustifolia*	China	KF765677	KF765693	KU710996	KU710956
*Cytospora eriobotryae*	IMI 136523*	*Eriobotrya japonica*	India	AY347327	NA	NA	NA
*Cytospora erumpens*	CFCC 50022	*Prunus padus*	China	MH933627	MH933661	MH933534	NA
	MFLUCC 16-0580*	Salix × fragilis	Russia	KY417733	KY417767	KY417699	KY417801
*Cytospora eucalypti*	CBS 144241	*Eucalyptus globulus*	USA	MG971907	NA	MG972056	NA
	LSEQ	*Sequoia sempervirens*	USA	AY347340	NA	NA	NA
*Cytospora eucalypticola*	ATCC 96150*	*Eucalyptus nitens*	Australia	AY347358	NA	NA	NA
	CMW 5309	*Eucalyptus grandis*	Uganda	AF260266	NA	NA	NA
*Cytospora eucalyptina*	CMW 5882	*Eucalyptus grandis*	Columbia	AY347375	NA	NA	NA
*Cytospora eugeniae*	CMW 7029	*Tibouchina* sp.	Australia	AY347364	NA	NA	NA
	CMW 8648	*Eugenia* sp.	Indonesia	AY347344	NA	NA	NA
*Cytospora euonymicola*	CFCC 50499*	*Euonymus kiautschovicus*	China	MH933628	MH933662	MH933535	MH933598
	CFCC 50500	*Euonymus kiautschovicus*	China	MH933629	MH933663	MH933536	MH933599
*Cytospora euonymina*	CFCC 89993*	*Euonymus kiautschovicus*	China	MH933630	MH933664	MH933537	MH933600
	CFCC 89999	*Euonymus kiautschovicus*	China	MH933631	MH933665	MH933538	MH933601
*Cytospora fraxinigena*	MFLUCC 14-0868*	*Euonymus kiautschovicus*	China	MH933631	MH933665	MH933538	MH933601
*Cytospora friesii*	CBS 194.42	*Abies alba*	Switzerland	AY347328	NA	NA	NA
*Cytospora fugax*	CXY 1381	NA	NA	KM034853	NA	NA	NA
*Cytospora germanica*	CXY 1322	*Elaeagnus oxycarpa*	China	JQ086563	JX524617	NA	NA
*Cytospora gigalocus*	CFCC 89620*	*Juglans regia*	China	KR045628	KR045708	KU710997	KU710957
	CFCC 89621	*Juglans regia*	China	KR045629	KR045709	KU710998	KU710958
*Cytospora gigaspora*	CFCC 50014	*Juniperus procumbens*	China	KR045630	KR045710	KU710999	KU710959
	CFCC 89634*	*Salix psammophila*	China	KF765671	KF765687	KU711000	KU710960
*Cytospora granati*	CBS 144237*	*Punica granatum*	USA	MG971799	NA	MG971949	NA
*Cytospora hippophaës*	CFCC 89639	*Hippophaë rhamnoides*	China	KR045632	KR045712	KU711001	KU710961
	CFCC 89640	*Hippophaë rhamnoides*	China	KF765682	KF765698	KF765730	KU710962
*Cytospora japonica*	CFCC 89956	*Prunus cerasifera*	China	KR045624	KR045704	KU710993	KU710953
	CFCC 89960	*Prunus cerasifera*	China	KR045625	KR045705	KU710994	KU710954
*Cytospora joaquinensis*	CBS 144235*	*Populus deltoides*	USA	MG971895	NA	MG972044	NA
*Cytospora junipericola*	MFLU 17-0882*	*Juniperus communis*	Italy	MF190125	MF190072	NA	NA
*Cytospora juniperina*	CFCC 50501*	*Juniperus przewalskii*	China	MH933632	MH933666	MH933539	MH933602
	CFCC 50503	*Juniperus przewalskii*	China	MH933634	MH933668	MH933541	MH933604
*Cytospora kantschavelii*	CXY 1383	*Populus maximowiczii*	China	KM034867	NA	NA	NA
***Cytospora kuanchengensis***	CFCC 52464*	***Castanea mollissima***	**China**	**MK432616**	**MK429886**	**MK442940**	**MK578076**
	**CFCC 52465**	***Castanea mollissima***	**China**	**MK432617**	**MK429887**	**MK442941**	**MK578077**
*Cytospora kunzei*	CBS 118556	*Pinus radiata*	South Africa	DQ243791	NA	NA	NA
*Cytospora leucosperma*	CFCC 89622	*Pyrus bretschneideri*	China	KR045616	KR045698	KU710988	KU710944
	CFCC 89894	*Pyrus bretschneideri*	China	KR045617	KR045699	KU710989	KU710945
***Cytospora leucostoma***	CFCC 50018	*Prunus serrulata*	China	MH933636	MH933670	MH933543	NA
	CFCC 50019	*Rosa helenae*	China	MH933637	MH933671	MH933544	NA
	CFCC 50021	*Prunus salicina*	China	MH933639	MH933673	MH933546	NA
	CFCC 50023	*Cornus alba*	China	KR045635	KR045715	KU711003	KU710964
	**CFCC 52461**	***Castanea mollissima***	**China**	**MK432624**	**MK429894**	**MK442948**	**NA**
	**CFCC 52462**	***Castanea mollissima***	**China**	**MK432625**	**MK429895**	**MK442949**	**NA**
*Cytospora longiostiolata*	MFLUCC 16-0628*	Salix × fragilis	Russia	KY417734	KY417768	KY417700	KY417802
*Cytospora longispora*	CBS 144236*	*Prunus domestica*	USA	MG971905	NA	MG972054	NA
*Cytospora lumnitzericola*	MFLUCC 17-0508*	*Lumnitzera racernosa*	Tailand	MG975778	MH253461	MH253457	MH253453
*Cytospora mali*	CFCC 50028	*Malus pumila*	China	MH933641	MH933675	MH933548	MH933606
	CFCC 50029	*Malus pumila*	China	MH933642	MH933676	MH933549	MH933607
*Cytospora melnikii*	MFLUCC 15-0851*	*Malus domestica*	Russia	KY417735	KY417769	KY417701	KY417803
	MFLUCC 16-0635	Populus nigra var. italica	Russia	KY417736	KY417770	KY417702	KY417804
*Cytospora mougeotii*	ATCC 44994	*Picea abies*	Norway	AY347329	NA	NA	NA
*Cytospora multicollis*	CBS 105.89T	Quercus ilex subsp. rotundifolia	Spain	DQ243803	NA	NA	NA
*Cytospora myrtagena*	CBS 116843*	*Tibouchiina urvilleana*	USA	AY347363	NA	NA	NA
	**CFCC 52454**	***Castanea mollissima***	**China**	**MK432614**	**MK429884**	**MK442938**	**MK578074**
	**CFCC 52455**	***Castanea mollissima***	**China**	**MK432615**	**MK429885**	**MK442939**	**MK578075**
*Cytospora nitschkii*	CMW 10180*	*Eucalyptus globulus*	Ethiopia	AY347356	NA	NA	NA
	CMW 10184	*Eucalyptus globulus*	Ethiopia	AY347355	NA	NA	NA
*Cytospora nivea*	CFCC 89641	*Elaeagnus angustifolia*	China	KF765683	KF765699	KU711006	KU710967
	CFCC 89643	*Salix psammophila*	China	KF765685	KF765701	NA	KU710968
*Cytospora oleicola*	CBS 144248*	*Olea europaea*	USA	MG971944	NA	MG972098	NA
*Cytospora palm*	CXY 1280*	*Cotinus coggygria*	China	JN411939	NA	NA	NA
*Cytospora parakantschavelii*	MFLUCC 15-0857*	Populus × sibirica	Russia	KY417738	KY417772	KY417704	KY417806
	MFLUCC 16-0575	*Pyrus pyraster*	Russia	KY417739	KY417773	KY417705	KY417807
*Cytospora parapersoonii*	T28.1*	*Prunus persica*	USA	AF191181	NA	NA	NA
*Cytospora parapistaciae*	CBS 144506*	*Pistacia vera*	USA	MG971804	NA	MG971954	NA
*Cytospora parasitica*	MFLUCC 15-0507*	*Malus domestica*	Russia	KY417740	KY417774	KY417706	KY417808
*Cytospora paratranslucens*	MFLUCC 15-0506*	Populus alba var. bolleana	Russia	KY417741	KY417775	KY417707	KY417809
	MFLUCC 16-0627	*Populus alba*	Russia	KY417742	KY417776	KY417708	KY417810
*Cytospora pini*	CBS 197.42	*Pinus sylvestris*	Switzerland	AY347332	NA	NA	NA
	CBS 224.52*	*Pinus strobus*	USA	AY347316	NA	NA	NA
*Cytospora pistaciae*	CBS 144238*	*Pistacia vera*	USA	MG971802	NA	MG971952	NA
*Cytospora platanicola*	MFLU 17-0327*	*Platanus hybrida*	Italy	MH253451	MH253452	MH253449	MH253450
*Cytospora platycladi*	CFCC 50504*	*Platycladus orientalis*	China	MH933645	MH933679	MH933552	MH933610
	CFCC 50505	*Platycladus orientalis*	China	MH933646	MH933680	MH933553	MH933611
	CFCC 50506	*Platycladus orientalis*	China	MH933647	MH933681	MH933554	MH933612
*Cytospora platycladicola*	CFCC 50038*	*Platycladus orientalis*	China	KT222840	MH933682	MH933555	MH933613
	CFCC 50039	*Platycladus orientalis*	China	KR045642	KR045721	KU711008	KU710973
*Cytospora plurivora*	CBS 144239*	*Olea europaea*	USA	MG971861	NA	MG972010	NA
*Cytospora populicola*	CBS 144240*	*Populus deltoides*	USA	MG971891	NA	MG972040	NA
*Cytospora populina*	CFCC 89644*	*Salix psammophila*	China	KF765686	KF765702	KU711007	KU710969
*Cytospora populinopsis*	CFCC 50032*	*Sorbus aucuparia*	China	MH933648	MH933683	MH933556	MH933614
	CFCC 50033	*Sorbus aucuparia*	China	MH933649	MH933684	MH933557	MH933615
*Cytospora predappioensis*	MFLUCC 17-2458*	*Platanus hybrida*	Italy	MG873484	MG873480	NA	NA
*Cytospora prunicola*	MFLU 17-0995*	*Prunus* sp.	Italy	MG742350	MG742351	MG742353	MG742352
*Cytospora pruinopsis*	CFCC 50034*	*Ulmus pumila*	China	KP281259	KP310806	KP310836	KU710970
	CFCC 50035	*Ulmus pumila*	China	KP281260	KP310807	KP310837	KU710971
*Cytospora pruinosa*	CFCC 50036	*Syringa oblata*	China	KP310800	KP310802	KP310832	NA
	CFCC 50037	*Syringa oblata*	China	MH933650	MH933685	MH933558	NA
*Cytospora prunicola*	MFLU 17-0995*	*Prunus* sp.	Italy	MG742350	MG742351	MG742353	MG742352
*Cytospora punicae*	CBS 144244	*Punica granatum*	USA	MG971943	NA	MG972091	NA
*Cytospora quercicola*	MFLU 17-0881	*Quercus* sp.	Italy	MF190129	MF190074	NA	NA
	MFLUCC 14-0867*	*Quercus* sp.	Italy	MF190128	MF190073	NA	NA
*Cytospora rhizophorae*	MUCC302	*Eucalyptus grandis*	Australia	EU301057	NA	NA	NA
*Cytospora ribis*	CFCC 50026	*Ulmus pumila*	China	KP281267	KP310813	KP310843	KU710972
	CFCC 50027	*Ulmus pumila*	China	KP281268	KP310814	KP310844	NA
*Cytospora rosae*	MFLU 17-0885	*Rosa canina*	Italy	MF190131	MF190076	NA	NA
*Cytospora rostrata*	CFCC 89909*	*Salix cupularis*	China	KR045643	KR045722	KU711009	KU710974
	CFCC 89910	*Salix cupularis*	China	KR045644	KR045723	KU711010	KU710975
*Cytospora rusanovii*	MFLUCC 15-0853	Populus × sibirica	Russia	KY417743	KY417777	KY417709	KY417811
	MFLUCC 15-0854*	*Salix babylonica*	Russia	KY417744	KY417778	KY417710	KY417812
*Cytospora salicacearum*	MFLUCC 16-0576	dead aerial branch	Russia	KY417747	KY417781	KY417713	KY417815
	MFLUCC 15-0509*	*Salix alba*	Russia	KY417746	KY417780	KY417712	KY417814
	MFLUCC 15-0861	Salix × fragilis	Russia	KY417745	KY417779	KY417711	KY417813
	MFLUCC 16-0587	NA	NA	KY417748	KY417782	KY417714	KY417816
*Cytospora salicicola*	MFLUCC 14-1052*	*Salix alba*	Russia	KU982636	KU982635	KU982637	NA
	MFLUCC 15-0866	*Salix alba*	Russia	KY417749	KY417783	KY417715	KY417817
*Cytospora salicina*	MFLUCC 15-0862*	*Salix alba*	Russia	KY417750	KY417784	KY417716	KY417818
	MFLUCC 16-0637	Salix × fragilis	Russia	KY417751	KY417785	KY417717	KY417819
***Cytospora schulzeri***	CFCC 50040	*Malus domestica*	China	KR045649	KR045728	KU711013	KU710980
	CFCC 50042	*Malus asiatica*	China	KR045650	KR045729	KU711014	KU710981
	**CFCC 52468**	***Castanea mollissima***	**China**	**MK432626**	**MK429896**	**MK442950**	**MK578084**
	**CFCC 52469**	***Castanea mollissima***	**China**	**MK432627**	**MK429897**	**MK442951**	**MK578085**
	**CFCC 52470**	***Castanea mollissima***	**China**	**MK432628**	**MK429898**	**MK442952**	**MK578086**
*Cytospora sibiraeae*	CFCC 50045*	*Sibiraea angustata*	China	KR045651	KR045730	KU711015	KU710982
	CFCC 50046	*Sibiraea angustata*	China	KR045652	KR045731	KU711015	KU710983
*Cytospora sophorae*	CFCC 50048	*Magnolia grandiflora*	China	MH820401	MH820394	MH820409	MH820397
	CFCC 89598	*Styphnolobium japonicum*	China	KR045654	KR045733	KU711018	KU710985
*Cytospora sophoricola*	CFCC 89595*	Styphnolobium japonicum var. pendula	China	KR045655	KR045734	KU711019	KU710986
	CFCC 89596	Styphnolobium japonicum var. pendula	China	KR045656	KR045735	KU711020	KU710987
*Cytospora sophoriopsis*	CFCC 89600*	*Styphnolobium japonicum*	China	KR045623	KP310804	KU710992	KU710951
*Cytospora sorbi*	MFLUCC 16-0631*	*Sorbus aucuparia*	Russia	KY417752	KY417786	KY417718	KY417820
*Cytospora sorbicola*	MFLUCC 16-0584*	*Acer pseudoplatanus*	Russia	KY417755	KY417789	KY417721	KY417823
	MFLUCC 16-0633	*Cotoneaster melanocarpus*	Russia	KY417758	KY417792	KY417724	KY417826
*Cytospora spiraeae*	CFCC 50049*	*Spiraea salicifolia*	China	MG707859	MG707643	MG708196	MG708199
	CFCC 50050	*Spiraea salicifolia*	China	MG707860	MG707644	MG708197	MG708200
*Cytospora tamaricicola*	CFCC 50507	*Rosa multifolora*	China	MH933651	MH933686	MH933559	MH933616
	CFCC 50508*	*Tamarix chinensis*	China	MH933652	MH933687	MH933560	MH933617
*Cytospora tanaitica*	MFLUCC 14-1057*	*Betula pubescens*	Russia	KT459411	KT459412	KT459413	NA
*Cytospora thailandica*	MFLUCC 17-0262*	*Xylocarpus moluccensis*	Thailand	MG975776	MH253463	MH253459	MH253455
	MFLUCC 17-0263	*Xylocarpus moluccensis*	Thailand	MG975777	MH253464	MH253460	MH253456
*Cytospora tibouchinae*	CPC 26333*	*Tibouchina semidecandra*	France	KX228284	KX228335	NA	NA
*Cytospora translucens*	CXY 1351	*Populus davidiana*	China	KM034874	NA	NA	NA
*Cytospora ulmi*	MFLUCC 15-0863*	*Ulmus minor*	Russia	KY417759	NA	NA	NA
*Cytospora ulmicola*	MFLUCC 18-1227*	*Ulmus pumila*	Russia	MH940220	MH940218	MH940216	NA
*Cytospora valsoidea*	CMW 4309*	*Eucalyptus grandis*	Indonesia	AF192312	NA	NA	NA
	CMW 4310	*Eucalyptus grandis*	Indonesia	AF192312	NA	NA	NA
*Cytospora variostromatica*	CBS 118086	*Eucalyptus grandis*	South Africa	AF260264	NA	NA	NA
	CMW 1240	*Eucalyptus grandis*	South Africa	AF260263	NA	NA	NA
	CMW 6766*	*Eucalyptus globulus*	Australia	AY347366	NA	NA	NA
*Cytospora vinacea*	CBS 141585*	*Vitis* interspecific hybrid ‘Vidal’	USA	KX256256	NA	NA	NA
*Cytospora viticola*	CBS 141586*	*Vitis vinifera* ’Cabernet Franc’	USA	KX256239	NA	NA	NA
*Cytospora xinglongensis*	CFCC 52458*	***Castanea mollissima***	**China**	**MK432622**	**MK429892**	**MK442946**	**MK578082**
	**CFCC 52459**	***Castanea mollissima***	**China**	**MK432623**	**MK429893**	**MK442947**	**MK578083**
*Cytospora xylocarpi*	MFLUCC 17-0251*	*Xylocarpus granatum*	Thailand	MG975775	MH253462	MH253458	MH253454
*Diaporthe vaccinii*	CBS 160.32	*Vaccinium macrocarpon*	USA	KC343228	NA	JQ807297	NA

## Results

### Phylogenetic analyses

**Figure 2.**
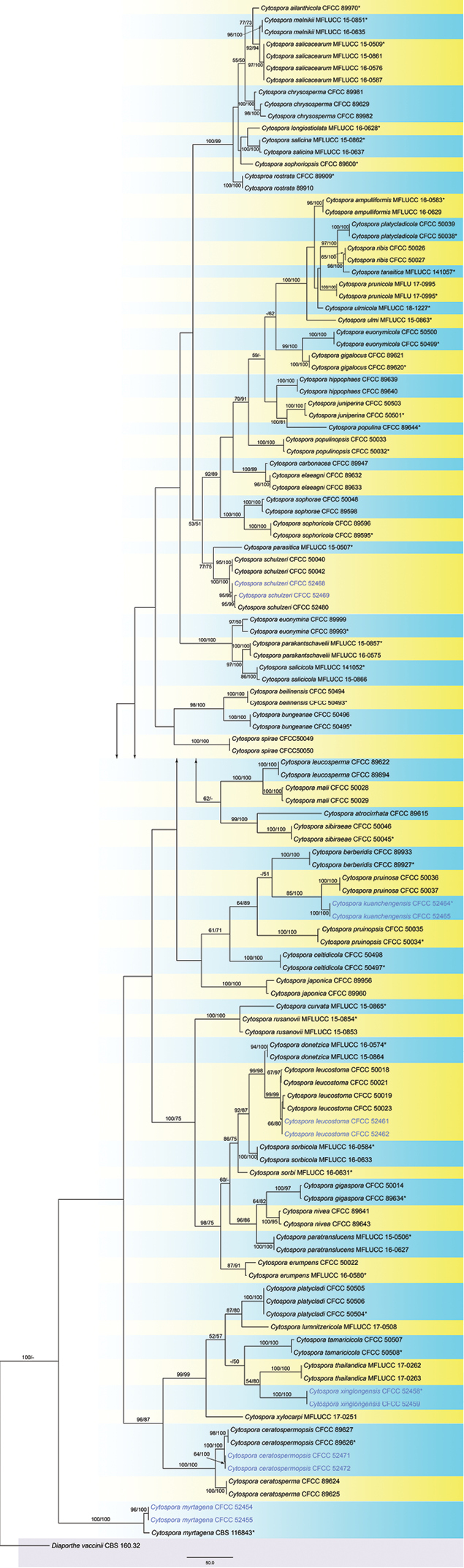
Maximum parsimony phylogram of *Cytospora* obtained from the combined matrix of ITS, LSU, ACT and RPB2 genes. Bootstrap value ≥ 50% for MP and ML analyses are presented at the first and second position. Scale bar = 200 nucleotide substitutions. The strains in the current study are in blue and ex-strains are marked with *.

The alignment based on the combined sequence dataset (ITS, LSU, ACT and RPB2) included 124 ingroup taxa and one outgroup taxon, comprising 2097 characters in the aligned matrix. Of these, 1375 characters were constant, 89 variable characters were parsimony-uninformative and 663 characters were parsimony informative. The MP analysis resulted in 14 equally most parsimonious trees and the first tree (TL = 3270, CI = 0.344, RI = 0.815, RC = 0.281) was present as in Fig. 2. The ML analysis yielded a tree with a likelihood value of ln: -18627.915604 and the following model parameters: alpha: 0.181328; Π(A): 0.246855, Π(C): 0.260898, Π(G): 0.272379 and Π(T): 0.219868. Isolates from *Castanea mollissima* formed six clades in Fig. 2, representing two undescribed species and four known species.

### Taxonomy

#### Cytospora ceratosp ermopsis

Taxon classificationFungiDiaporthalesValsaceae

C.M. Tian & X.L. Fan, Persoonia 45: 19. 2020

990B2D8E-95BC-5250-884C-64AF73BCEF11

Figure 3

##### Description.

Sexual morph: Ascostromata immersed in the bark, erumpent through the surface of bark, scattered, (350–)550–900(–1300) µm diam., with 15–40 perithecia arranged circularly or irregularly. Conceptacle absent. Ectostromatic disc black, usually surrounded by tightly ostiolar necks, circular to ovoid, (180–)240–410(–450) µm diam. Ostioles black, at the same level as the disc or slightly above, concentrated, dark brown to black, arranged circularly in a disc, (55–)60–85(–110) µm diam. Perithecia dark brown, flask-shaped to spherical, arranged circularly or irregularly, (255–)280–350(–420) µm diam. Asci clavate to elongate obovoid, 8-spored, (20.5–)27–35.5(–43) × (4–)4.5–6.5(–8) μm (x̄= 31.2 × 5.6 μm). Ascospores biseriate, elongate-allantoid, thin-walled, hyaline, aseptate, (5.8–)7.5–9.2(–11.5) × (3–)3.2–4.8(–5.5) μm (x̄ = 8.6 × 4.1 μm). Asexual morph: Pycnidial stromata ostiolated, immersed in bark, scattered, erumpent through the surface of bark, discoid to conical, with multiple locules. Conceptacle absent. Ectostromatic disc light brown to grey, circular to ovoid, (230–)280–360(–480) µm diam., with one ostiole per disc. Ostiole in the centre of the disc, dark grey to black, conspicuous, at the same level as the disc, (60–)75–110(–135) μm diam. Locule numerous, arranged circularly or elliptically with independent walls, (300–)350–600(–950) µm diam. Peridium comprising few layers of cells of textura angularis, with innermost layer brown, outer layer brown to dark brown. Conidiophores hyaline, branched or not, thin walled, filamentous. Conidiogenous cells enteroblastic polyphialidic, (6.5–)8.5–15.5(–18) × 1.5–2.5 μm (x̄ = 12.2 × 1.9 μm). Conidia hyaline, allantoid, smooth, aseptate, thin-wall, (4.5–)5–6.5(–7) × 1–1.5 μm (x̄ = 5.9 × 1.3 μm).

##### Culture characters.

On PDA at 25 °C in darkness. Cultures are initially white, becoming olivaceous buff in centre after 7 d and finally olivaceous at 30 d. The colony is flat, thin with a felt and tight texture in centre. Pycnidia distributed irregularly on medium surface.

##### Specimens examined.

China, Hebei Province, Chengde City, Xinglong County, chestnut plantation, 40°24'32"N, 117°27'56"E, on branches of *Castanea mollissima*, 11 October 2017, N. Jiang (BJFC-S1699, living culture CFCC 52471 from the ascospore; BJFC-S1700, living culture CFCC 52472 from the conidium).

##### Notes.

Fresh specimens with both sexual and asexual morphs were collected from cankered branches of *Castanea mollissima* and two isolates were obtained from the ascospore and conidium, respectively. Phylogenically, the two isolates were close to *Cytospora ceratospermopsis* represented by CFCC 89626 and CFCC 89627 (Fig. 2). We compared their sequences and found no differences in LSU and RPB2, but 2 bp differences in ITS and 3 bp differences in ACT. Fan et al. (2020) reported the asexual morph of *Cytospora ceratospermopsis* from *Juglans regia* in China with conidial size in 4.5–6 × 1–1.5 μm, which is exactly matched with the asexual characters observed in the present study. Hence, we described the asexual morph of *Cytospora ceratospermopsis* in its sexual morph for the first time and reported a new host, *Castanea mollissima*.

**Figure 3.**
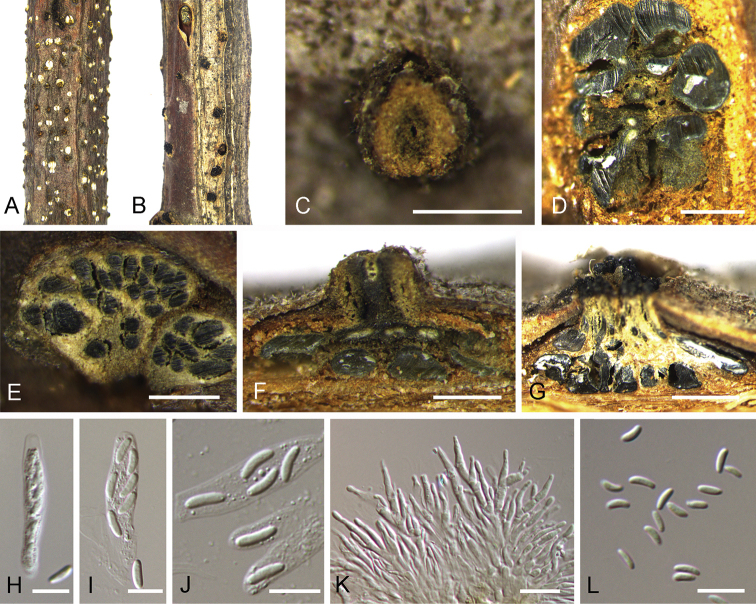
*Cytospora ceratospermopsis* on *Castanea mollissima* (BJFC-S1699, BJFC-S1700). **A, C** Habit of conidiomata on branches **B** habit of ascomata on branches **D** transverse section of conidiomata **E** transverse section of ascomata **F** longitudinal section through conidiomata **G** longitudinal section through ascomata **H, I** asci **J** ascospores **K** conidiogenous cells with attached conidia **L** conidia. Scale bars: 500 μm (**C–G**), 10 μm (**H–L**).

#### Cytospora kuanchengensis

Taxon classificationFungiDiaporthalesValsaceae

C.M. Tian & N. Jiang sp. nov.

2BB85219-15AA-55BA-8D48-E0C738D2DA36

829514

Figure 4

##### Diagnosis.

*Cytospora kuanchengensis* can be distinguished from *C. oleicola* and *C. pruinose* by longer conidia.

##### Etymology.

Named after the county where it was collected, Kuancheng County.

##### Description.

Sexual morph: not observed. Asexual morph: Pycnidial stromata ostiolated, immersed in bark, scattered, erumpent through the surface of bark, discoid, with multiple locules. Conceptacle black, circular surrounded stromata. Ectostromatic disc black, circular to ovoid, (350–)455–540(–575) µm diam., with 1–7 ostiole per disc. Ostioles black, at the same level as the disc, (40–)60–85(–115) μm diam. Locule numerous, arranged circularly or elliptically with independent walls, (285–)355–520(–605) µm diam. Peridium comprising few layers of cells of textura angularis, with innermost layer brown, outer layer brown to dark brown. Conidiophores hyaline, unbranched, thin walled, filamentous. Conidiogenous cells enteroblastic polyphialidic, (6.5–)8.5–11(–15) × 1–1.5 μm (x̄ = 9.8 × 1.3 μm). Conidia hyaline, allantoid, smooth, aseptate, thin-walled, (5.5–)6–7.5(–8) × 1–2 μm (x̄ = 6.9 × 1.6 μm).

##### Culture characters.

On PDA at 25 °C in darkness. Cultures are initially white, producing pale brown pigment after 10 d. The colony is flat, felt-like, with concentric circular texture. Pycnidia distributed irregularly on medium surface.

##### Specimens examined.

China, Hebei Province, Chengde City, Kuancheng County, chestnut plantation, 40°38'37"N, 118°27'54"E, on branches of *Castanea mollissima*, 13 October 2017, N. Jiang (***holotype***BJFC-S1695, ex-type living culture CFCC 52464; ***paratype***BJFC-S1696, living culture CFCC 52465).

##### Notes.

*Cytospora kuanchengensis* is associated with canker disease of *Castanea mollissima* in China. *Cytospora kuanchengensis* differs from its phylogenetically closely species, *C. pruinosa*, by ITS and ACT loci (7/470 in ITS and 21/245 in ACT). Morphologically, *C. kuanchengensis* has slightly larger conidia than *C. pruinose* (5.5–8 × 1–2 μm in *Cytospora kuanchengensis* vs. 5–7.5 × 1–1.5 μm in *C. pruinosa*) (Fan et al. 2020).

**Figure 4.**
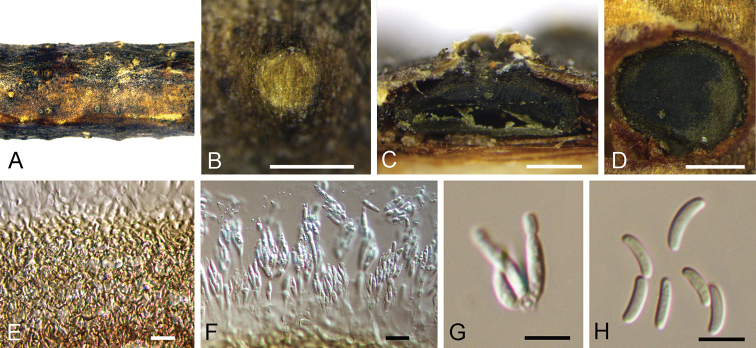
*Cytospora kuanchengensis* on *Castanea mollissima* (BJFC-S1695). **A, B** Habit of conidiomata on branches **C** longitudinal section through conidiomata **D** transverse section of conidiomata **E** peridium **F, G** conidiogenous cells attached with conidia **H** conidia. Scale bars: 500 μm (**B–D**), 10 μm (**E–G**), 5 μm (**H**).

#### Cytospora leucostoma

Taxon classificationFungiDiaporthalesValsaceae

(Pers.) Sacc., Michelia 2(7): 264. 1881.

3D4086B0-49AA-50B7-A2BE-60091113DAAA

Figure 5

##### Description.

Sexual morph: not observed. Asexual morph: Pycnidial stromata ostiolated, immersed in bark, scattered, erumpent through the surface of bark, with multiple locules. Conceptacle black. Ectostromatic disc black, circular to ovoid, (150–)250–300(–375) µm diam., with one ostiole per disc. Ostioles black, at the same level as the disc, (40–)50–85(–115) μm diam. Locule numerous, arranged circularly or elliptically with independent walls, (550–)700–1200(–1350) µm diam. Peridium comprising few layers of cells of textura angularis, with innermost layer brown, outer layer brown to dark brown. Conidiophores hyaline, unbranched, thin walled, filamentous. Conidiogenous cells enteroblastic polyphialidic, (7.5–)9.5–21(–22.5) × 1–1.5 μm (x̄ = 15.2 × 1.3 μm). Conidia hyaline, allantoid, smooth, aseptate, thin-walled, (3.5–)4.5–5.5(–7) × 1–1.5 μm (x̄ = 4.9 × 1.3 μm).

##### Specimens examined.

China, Hebei Province, Chengde City, Kuancheng County, chestnut plantation, 40°38'37"N, 118°27'5"E, on branches of *Castanea mollissima*, 13 October 2017, N. Jiang (BJFC-S1697, living culture CFCC 52461; BJFC-S1698, living culture CFCC 52462).

##### Notes.

*Cytospora leucostoma* is a common species causing canker disease on Rosaceae in China (Teng 1963, Tai 1979, Wei 1979, Fan et al. 2020). In this study, fresh specimens were collected from diseased branches of the Chinese chestnut for the first time and identified as *Cytospora leucostoma*, based on strictly matched asexual morph (4–5.5 × 1–2 μm from *Castanea mollissima* in this study vs. 4.5–5.5 × 1–1.5 μm from multiple specimens in Fan et al. 2020) and phylogenic analysis (Fig. 2).

**Figure 5.**
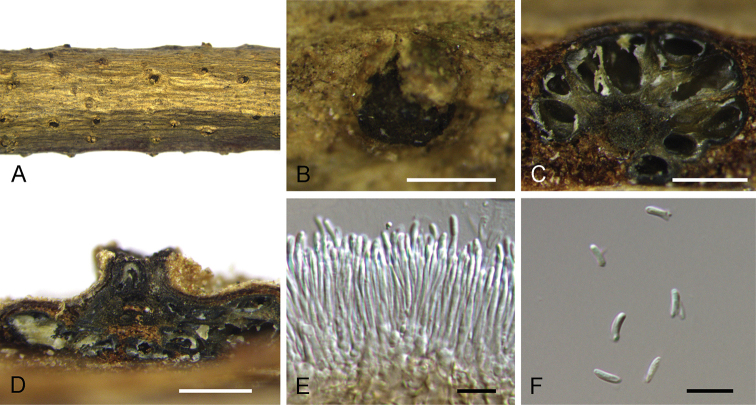
*Cytospora leucostoma* on *Castanea mollissima* (BJFC-S1697). **A, B** Habit of conidiomata on branches **C** transverse section of conidiomata **D** longitudinal section through conidiomata **E** conidiogenous cells attached with conidia **F** conidia. Scale bars: 500 μm (**B–D**), 10 μm (**E, F**).

#### Cytospora myrtagena

Taxon classificationFungiDiaporthalesValsaceae

(G.C. Adams & M.J. Wingf.) G.C. Adams & Rossman, IMA Fungus 6 (1): 147. 2015.

8A67297D-DD92-575B-B7B2-A24925C785AD

Figure 6

##### Description.

Sexual morph: not observed. Asexual morph: Pycnidial stromata pulvinate, immersed in bark, scattered, erumpent through the surface of bark. Conceptacle absent. Ostiole dark grey to black, conspicuous, at the same level as the disc, (50–)65–75(–82) μm diam. Locules undivided, circular to ovoid, (430–)550–720(–810) µm diam. Peridium comprising few layers of cells of textura angularis, with innermost layer brown, outer layer brown to dark brown. Conidiophores hyaline, unbranched, thin-walled, filamentous. Conidiogenous cells enteroblastic polyphialidic, (6.5–)8.4–12.5(–15.3) × 0.9–1.4 μm (x̄ = 10.2 × 1.2 μm). Conidia hyaline, allantoid, smooth, aseptate, thin-walled, (3.2–)3.4–5.4(–6.2) × 1–1.5 μm (x̄ = 4.7 × 1.3 μm).

##### Culture characters.

On PDA at 25 °C in darkness. Cultures are initially white, becoming olivaceous buff in centre after 7 d and finally olivaceous at 30 d. The colony is flat, thin with a felt and tight texture in centre. Pycnidia distributed irregularly on medium surface.

##### Specimens examined.

China, Shaanxi Province, Ankang City, Xiangxidong forest park, 32°40'33"N, 109°18'57"E, on stem barks of *Castanea mollissima*, 1 July 2017, N. Jiang (BJFC-S1704, living culture CFCC 52454; BJFC-S1705, living culture CFCC 52455).

##### Notes.

*Cytospora myrtagena* was introduced from *Eucalyptus* and *Tibouchina* in America and Indonesia (Adams et al. 2005). Two ITS sequences of *Cytospora myrtagena* were available, AY347363 from the type strain CBS 116843 and AY347380 from CBS 117013. However, there are 14 bp differences between AY347363 and AY347380. *Cytospora tibouchinae* was introduced as a phylogenically close species to *Cytospora myrtagena* (Suppl. material 1: Fig. S1), with 21 bp differences to CBS 116843 and 14 bp bp differences to CBS 117013 (Crous et al. 2016). Two isolates from *Castanea mollissima* in the present study were close to *Cytospora myrtagena* and *Cytospora tibouchinae* (Suppl. material 1: Fig. S1), with 22 bp differences to CBS 116843, 15 bp differences to CBS 117013 and 6 bp differences to *Cytospora tibouchinae*. Morphologically, they have similar conidial sizes (3.4–5.4 × 1–1.5 μm in BJFC-S1704 vs. 3–4 × 1 μm in *C. myrtagena* vs. 3–4 × 1.5–2 μm in *C. tibouchinae*) (Adams et al. 2005, Crous et al. 2016). Hence, it is hard to identify our isolates to *C. myrtagena* or *C. tibouchinae*, for the large differences between two ITS sequences in *C. myrtagena* provided by Adams et al. (2005) and absence of ACT and RPB2 loci in *C. myrtagena* and *C. tibouchinae*. We give the name *Cytospora myrtagena* to our isolates provisionally, and hope for more studies on this species.

**Figure 6.**
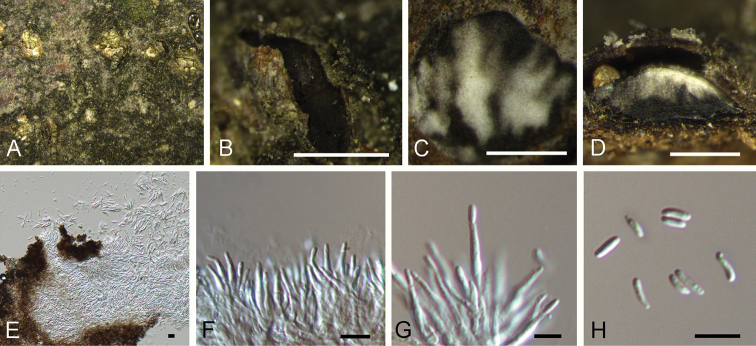
*Cytospora myrtagena* on *Castanea mollissima* (BJFC-S1704). **A, B** Habit of conidiomata on branches **C, E** transverse section of conidiomata **D** longitudinal section through conidiomata **F, G** conidiogenous cells attached with conidia **H** conidia. Scale bars: 500 μm (**B–D**), 5 μm (**E, G**), 10 μm (**H**).

#### Cytospora schulzeri

Taxon classificationFungiDiaporthalesValsaceae

Sacc. & P. Syd., Syll. fung. (Abellini) 14(2): 918. 1899.

17EB9794-D05F-5D4A-A3D3-7DDD2A3E30A1

Figure 7

##### Description.

Sexual morph: not observed. Asexual morph: Pycnidial stromata ostiolated, immersed in bark, scattered, erumpent through the surface of bark, flat, discoid, with multiple locules. Conceptacle absent. Ectostromatic disc brown, circular to ovoid, (250–)300–400(–475) µm diam., with 1–5 ostiole per disc. Ostioles black, at the same level as the disc, (40–)50–85(–115) μm diam. Locule numerous, arranged circularly with common walls, (600–)700–1500(–1750) µm diam. Peridium comprising a few layers of cells of textura angularis, with innermost layer brown, outer layer brown to dark brown. Conidiophores hyaline, unbranched, thin walled, filamentous. Conidiogenous cells enteroblastic polyphialidic, (6.5–)8.5–18.5(–21) × 1–2 μm (x̄ = 13.1 × 1.6 μm). Conidia hyaline, allantoid, smooth, aseptate, thin-walled, (3.5–)4.5–6.5(–7) × 1–1.5 μm (x̄ = 5.2 × 1.3 μm).

##### Specimens examined.

China, Hebei Province, Chengde City, Kuancheng County, chestnut plantation, 40°38'37"N, 118°27'54"E, on branches of *Castanea mollissima*, 13 October 2017, N. Jiang (living culture CFCC 52468; BJFC-S1702, living culture CFCC 52469; BJFC-S1703, living culture CFCC 52470).

##### Notes.

*Cytospora schulzeri* is a common species causing apple canker disease in China (Teng 1963, Tai 1979, Wei 1979, Zhuang 2005, Fan et al. 2020). In this study, fresh specimens were collected from diseased branches of chestnut trees and identified as *Cytospora schulzeri*, based on the strictly matched asexual morph (4.5–6.5 × 1–2 μm from *Castanea mollissima* in this study vs. 4–7 × 1–1.5 μm from multiple specimens in Fan et al. (2020)) and phylogenic analysis (Fig. 2).

**Figure 7.**
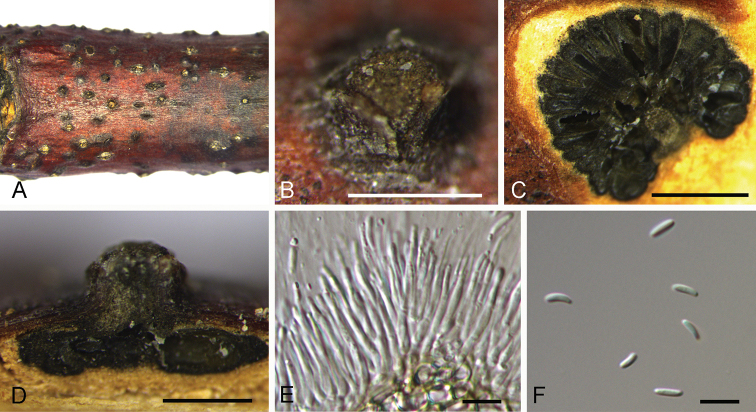
*Cytospora schulzeri* on *Castanea mollissima* (BJFC-S1702). **A, B** Habit of conidiomata on branches **C** transverse section of conidiomata **D** longitudinal section through conidiomata **E** conidiogenous cells attached with conidia **F** conidia. Scale bars: 500 μm (**B–D**), 10 μm (**E, F**).

#### Cytospora xinglongensis

Taxon classificationFungiDiaporthalesValsaceae

C.M. Tian & N. Jiang sp. nov.

D2D4ECD5-0D36-53AC-B6D1-29F9A3AA6879

829517

Figure 8

##### Diagnosis.

*Cytospora xinglongensis* can be distinguished from *C. californica* and *C. eucalypti* by longer conidia.

##### Etymology.

Named after the county where it was collected, Xinglong County.

##### Description.

Sexual morph: not observed. Asexual morph: Pycnidial stromata immersed in bark, erumpent through the surface of bark, discoid, with a solitary undivided locule. Conceptacle black, circular surrounded stromata. Ostiole inconspicuous. Locules undivided, circular to ovoid, (480–)540–685(–755) µm diam. Conidiophores hyaline, unbranched. Peridium comprising a few layers of cells of textura angularis, with innermost layer brown, outer layer brown to dark brown. Conidiogenous cells enteroblastic polyphialidic, (4.5–)6.5–8.5(–12) × 1–1.5 μm (x̄ = 7.4 × 1.3 μm). Conidia hyaline, allantoid, eguttulate, smooth, aseptate, thin-walled, (7.5–)8.5–9.5(–10.5) × 1–1.5 μm (x̄ = 8.9 × 1.3 μm).

##### Culture characters.

On PDA at 25 °C in darkness. Cultures are white. The colony is flat, thin with a uniform texture, lacking aerial mycelium. Pycnidia distributed uniformly on medium surface.

##### Specimens examined.

China, Hebei Province, Chengde City, Xinglong County, chestnut plantation, 40°24'32"N, 117°28'56"E, on branches of *Castanea mollissima*, 11 October 2017, N. Jiang (***holotype***BJFC-S1706, ex-type living culture CFCC 52458; ***paratype***BJFC-S1707, living culture CFCC 52459).

##### Notes.

*Cytospora xinglongensis* is associated with canker disease of *Castanea mollissima* in China. *Cytospora xinglongensis* can be distinguished from its phylogenetically closely species *C. thailandica* by having much longer conidia (8.5–9.5 μm in *C. xinglongensis* vs. 3.3–4 μm in *C. thailandica*) (Norphanphoun et al. 2018). In addition, *Cytospora xinglongensis* differs from *C. thailandica* by ITS, ACT and RPB2 loci (16/470 in ITS, 22/245 in ACT and 52/726 in RPB2).

**Figure 8.**
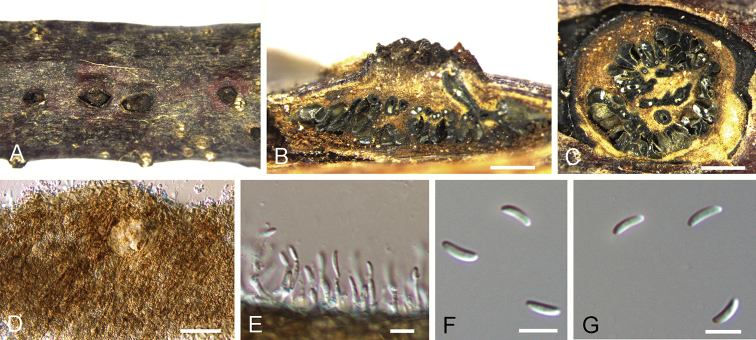
*Cytospora xinglongensis* on *Castanea mollissima* (BJFC-S1706). **A** Habit of conidiomata on branches **B** longitudinal section through conidiomata **C** transverse section of conidiomata **D** peridium **E** conidiogenous cells attached with conidia **F, G** conidia. Scale bars: 500 μm (**B, C**), 10 μm (**E–G**).

## Discussion

In the present study, an important fruit tree species, *Castanea mollissima* was investigated and *Cytospora* canker was found as a commom disease in plantations in Hebei Province. Identification was conducted based on 13 isolates from fruiting bodies using both morphological and molecular methods. As a result, six *Cytospora* species were confirmed. *Cytospora kuanchengensis* and *C. xinglongensis* are introduced as new species, *C. ceratospermopsis*, *C. leucostoma*, *C. myrtagena* and *C. schulzeri* are firstly reported on *Castanea mollissima*.

**Figure 9.**
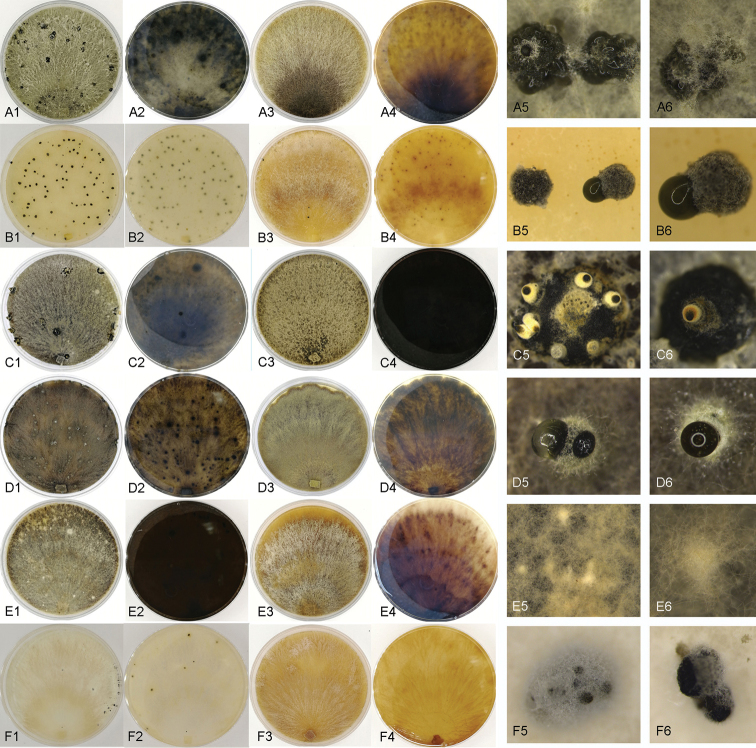
Cultures of *Cytospora* species from *Castanea mollissima* after 1 month at 25 °C. **A***C. myrtagena***B***C. kuanchengensis***C***C. ceratospermopsis***D***C. leucostoma***E***C. xinglongensis***F***C. schulzeri***A1–G2** cultures on PDA **A3–G4** cultures on MEA **A5–G6** fruiting bodies or hyphal masses produced on cultures

These six chestnut *Cytospora* species can be easily distinguished using DNA sequences of single ITS sequence or combined sequences of ITS, LSU, ACT and RPB2 (Fig. 2; Suppl. material 1: Fig. S1). In addition, colonies on PDA and MEA of these six species are also different (Fig. 9). *Cytospora xinglongensis* never produce fruiting bodies on PDA or MEA, while the other five species form conidiomata in one month (Fig. 9). Morphologically, *Cytospora xinglongensis* has obviously longer conidia than others. However, the conidial dimension can hardly distinguish *C. ceratospermopsis*, *C. kuanchengensis*, *C. leucostoma*, *C. myrtagena* and *C. schulzeri*.

Dar and Rai reported *Cytospora* diseases on *Castanea sativa* in India, causing perennial cankers on stems and branches (Dar and Rai 2014). The *Cytospora* isolates were identified mainly based on ITS sequence data, which were introduced as a new species named *Cytospora castaneae* (wrongly wrriten as *Cytospora castanae* in the original paper) (Dar and Rai 2014). However, further study is required to confirm the species position within the genus, including detailed morphogical features and sequences of high quality.

Cytospora canker is a common disease on chestnut trees, but there are few formal reports. In China, this disease is known amongst phytopathologists, but no-one conducted accurate identifications. Hence, this paper is the first formal report of Cytospora chestnut canker in China. From our investigations of chestnut diseases in China, *Cytospora* species are closely associated with canker diseases in chestnut plantations. In most cases, they infect twigs or small branches, causing necrotic lesions (Fig. 1A), finially forming fruiting bodies on dead tissues (Fig. 1D). However, *Cytospora myrtagena* was discovered on stems of a 15-year-old chestnut tree, causing typical *Cytospora* canker symptoms. More works should be conducted on the newly emerging pathogens from several aspects.

As the species concept of *Cytospora* has been improved a lot by using molecular data (Yang et al. 2015, Lawrence et al. 2017, Norphanphoun et al. 2017, 2018, Jayawardena et al. 2019, Fan et al. 2020), many Cytospora canker diseases and new species have been discovered and reported in recent years. Further studies are, however, now required to confirm their pathogenicity.

## Supplementary Material

XML Treatment for Cytospora ceratosp ermopsis

XML Treatment for Cytospora kuanchengensis

XML Treatment for Cytospora leucostoma

XML Treatment for Cytospora myrtagena

XML Treatment for Cytospora schulzeri

XML Treatment for Cytospora xinglongensis

## Supplementary materials

Supplementary material 1 Figure S1 Data type: (phylogram of *Cytospora*) Explanation note: Phylogram of *Cytospora* obtained from the ITS gene. https://binary.pensoft.net/file/371661This dataset is made available under the Open Database License (http://opendatacommons.org/licenses/odbl/1.0/). The Open Database License (ODbL) is a license agreement intended to allow users to freely share, modify, and use this Dataset while maintaining this same freedom for others, provided that the original source and author(s) are credited.Ning Jiang, Qin Yang, Xin-Lei Fan, Cheng-Ming Tian
