# Supplementary figures and images for: Identification of six Cytospora species on Chinese chestnut in China
Source: MycoKeys. 2020 Jan 13;62:1–25. doi: 10.3897/mycokeys.62.47425 (PMC6971133; doi:10.3897/mycokeys.62.47425)

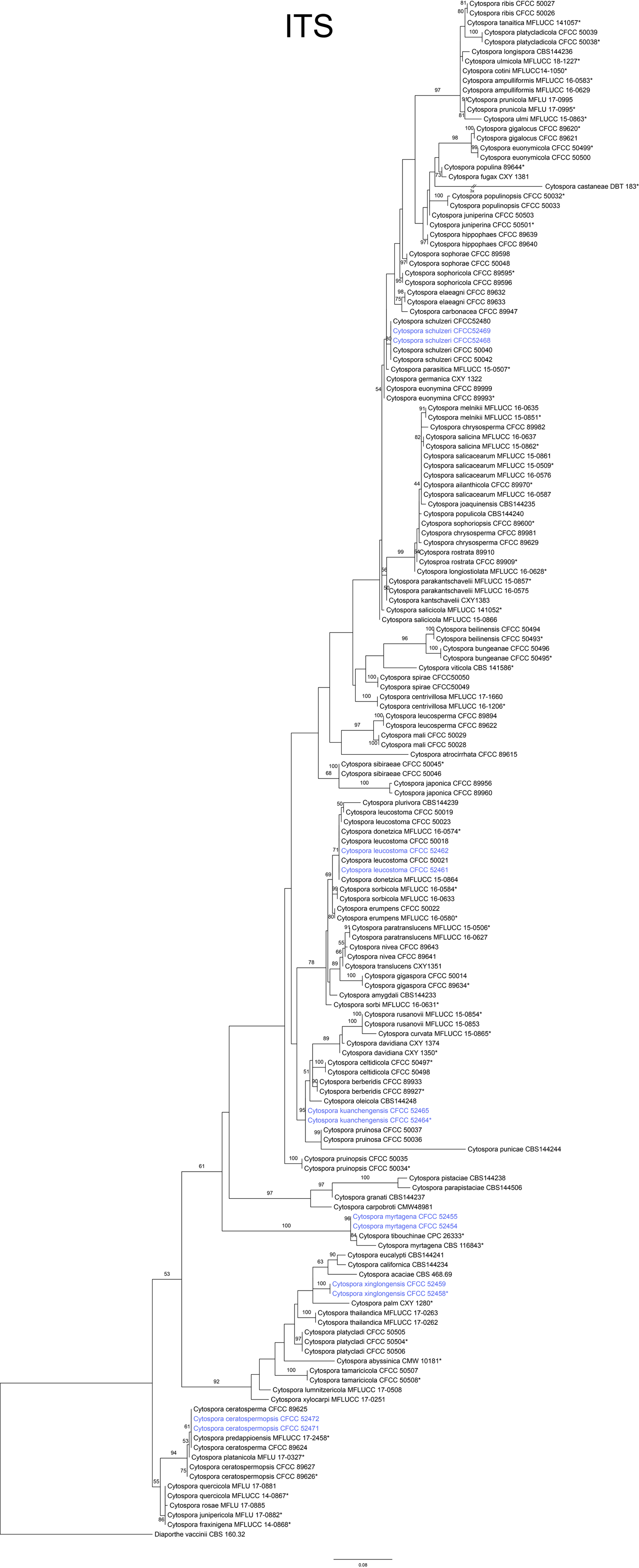

Supplement: Supplementary material 1 [file mycokeys-62-001-s001.tif]
